# Sampling Design and Sample Processing Affect Soil Biodiversity Assessments

**DOI:** 10.1111/1755-0998.70113

**Published:** 2026-02-24

**Authors:** Meirong Chen, Olesya Dulya, Vladimir Mikryukov, Ovidiu Copot, Martin Metsoja, Leho Tedersoo

**Affiliations:** ^1^ Institute of Ecology and Earth Sciences University of Tartu Tartu Estonia; ^2^ Mycology and Microbiology Center University of Tartu Tartu Estonia

**Keywords:** environmental DNA, high‐throughput sequencing, molecular artefact, PacBio, sample pooling, soil microorganisms

## Abstract

Biodiversity surveys require an appropriate sampling design for optimal performance and comparability across space and time and across studies. Based on PacBio and Illumina amplicon sequencing of animals, bacteria and fungi, we assessed and compared various soil sampling designs from widely used continental and global metabarcoding‐based biodiversity projects. Sampling designs revealed up to 27‐fold, 6‐fold and 15‐fold differences in biodiversity estimates for animals, bacteria and fungi, respectively. Taxonomic coverage depended mostly on the number of subsamples but not sampling area within the 347–1790 m^2^, 428–1924 m^2^, 327–1790 m^2^ plot size range for animals, bacteria and fungi, respectively. Additional sampling of subsoil did not add significantly to diversity estimates of animals and fungi, although there was a slight positive effect on bacteria. Both soil pooling (compositing subsamples before DNA extraction) and DNA pooling (combining DNA extracts prior to PCR) reduced differences between sampling designs by decreasing diversity estimates in designs with many subsamples while increasing them in designs with fewer subsamples. However, pooling did not eliminate the influence of sampling factors (soil depth, sampling area and sample size). DNA pooling outperformed soil pooling in inventorying animals and fungi but not bacteria. Pooling had no effect on recovering rare biological species or sequencing artefacts. Soil pooling saved from 79.5% to 98.3% of labour and analytical costs compared with no pooling, depending on the number of pooled subsamples. The remarkable impact of sampling design on community diversity and composition should be considered during data collection and meta‐analyses compiling data from different sampling designs.

## 1 Introduction

Determining a proper sampling design is a fundamental issue in biodiversity surveys, which relates to research questions, target organisms and resources (Bruckner et al. 2000; Querner and Bruckner 2010; Ranjard et al. 2003). Suboptimal sampling designs may bias biodiversity estimates, depending on the spatial pattern of sampling, sample size, sample volume and sampling area (Bell et al. 2005; Chase and Knight 2013; Kang and Mills 2006; MacArthur 1965; Rahbek 2005; Tan et al. 2017). Furthermore, 95% of sampling strategies are non‐reproducible due to subjectivity or insufficient description of the site selection or sampling process (Dickie et al. 2018; Whitcomb and Stutz 2007).

The largely stochastic community assembly, spatial aggregation, and enormous taxonomic richness make the selection of sampling designs for soil microbial diversity research more complicated than for macroorganisms (Fukami 2010; Green 1979; Kang and Mills 2006; Lemoine et al. 2023; Lücking et al. 2021). Appropriate sampling is particularly relevant in soil metabarcoding surveys due to the patchy distribution of species and our capacity to sample only a tiny fraction of the community. Research teams have reached different trade‐offs between sampling comprehensiveness and use of time in the field and lab and financial resources (Bach et al. 2018; Engel et al. 2012; Fierer and Jackson 2006; Guerra et al. 2021), but these decisions are typically unguided by the real data or power analyses.

To reduce the laboratory costs while maintaining representativeness, sampling designs involving subsampling are common in molecular ecology. The subsamples are usually pooled before or after DNA extraction to reduce the costs of sample processing and molecular analyses (Dickie et al. 2018). However, sample pooling has raised several technical concerns about equal subsample quality and quantity and overall dilution. Negative impacts of soil and DNA sample pooling on diversity estimates have been reported in ARISA (Automated ribosomal intergenic spacer Analysis) and T‐RFLP (Terminal restriction fragment length polymorphism) for bacteria, fungi and animals (Engel et al. 2012; Manter et al. 2010; Osborne et al. 2011). Studies based on high‐throughput sequencing (HTS) technologies that provide a higher resolution in species identification also show that the pooling effect varies among taxonomic groups, habitats, and diversity measures. In particular, soil pooling has caused loss of fungal species richness estimates but not on the Shannon diversity index (Song et al. 2015). Pooled samples enabled detection of fewer arthropods from soil (Porter et al. 2019). It is crucial to understand how different sampling design features—such as sampled area, sample number, depth and subsample pooling—affect diversity estimates of soil organisms. This would provide a source for information‐driven decisions about an optimal sampling design considering sampling representativeness, time and budgetary constraints.

In this study, we assessed the effects of sampling design and pooling strategies in recovering the richness of soil‐inhabiting bacteria, fungi and animals based on PacBio and Illumina amplicon sequencing. The study had three principal aims: (1) to evaluate the relative performance of existing, broadly used sampling designs in recovering biodiversity of different organism groups; (2) to estimate the effect of pooling either soil before DNA extraction or DNA extracts before the PCR step on species recovery; and (3) to provide information for decision making when considering sampling design and analytical strategies related to pooling. We expected that sampling designs with more subsamples covering a larger area would reveal more species (Green et al. 2004), especially in soil animals, that typically have patchy distribution (Berg 2012). We also suggested that the effect of pooling either soil or DNA depends on sampling design. In particular, we expected a stronger species loss for designs with larger sample sizes due to the dilution effect (Manter et al. 2010; Morita and Akao 2021). We also calculated the richness and Shannon diversity index at various sequencing depths to determine whether the resources saved by sample pooling could be reallocated to achieve greater sequencing depth, thereby increasing the probability of detecting low‐abundance species.

## 2 Methods

### 2.1 Sampling Designs and Field Work

**FIGURE 1:**
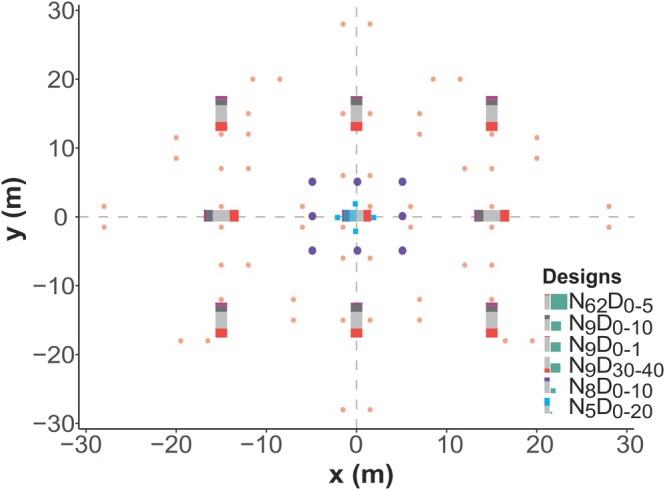
The scheme of sampling designs. Colours indicate different sampling designs. In legend symbols, the green rectangle is proportional to the sampling area and colour shadow in a grey bar denotes sampling depth. The columns in the map show the sampling depth of designs at overlapping core positions.

We conducted soil microbiome surveys in three natural forest sites growing on luvisols in Estonia (Tartu county). The Järvselja site (58°16′ N, 27°19′ E) represents an old‐growth (140 years) mixed forest dominated by *Tilia cordata* and *Picea abies* . The Kambja site (58°14′ N, 26°42′ E) is a *Betula pendula* and *Picea abies* dominated 120‐year‐old forest. The Vasula site (58°28′ N, 26°44′ E) is a dense *Quercus robur* and *Fraxinus excelsior* dominated woodland (140 years). In each forest site, we located a relatively homogeneous 10,000 m^2^ area and established a randomly selected point at least 3 m distant from the nearest tree (DBH > 10 cm) with a uniform microtopography within 1 m radius. Then we delimited the maximum sampling area (2500 m^2^) around this central core. These points represented plot centres for all compared sampling designs (Table 1, Figure 1). The samples were collected following the original protocols (see the referenced works in Table 1), using either a 50‐mm PVC pipe, a sharp knife or a teaspoon. In each site, soil sampling was performed in dry weather on a single day in July 2021. To partition the effects of the sampling area and sample size, we modified the *N*_40_ *D*_0–5A_ (40 subsamples of 0–5 cm depth in 2500 m^2^) original design (Tedersoo et al. 2021) by increasing the number of subsamples to 62 (*N*_62_ *D*_0–5_) or reducing the sampling area to 1400 m^2^ (*N*_40_ *D*_0–5B_). For the *N*_9_ *D*_0–10_ design used for Soil BON (Guerra et al. 2021), we collected four additional soil cores for the OTU richness estimates in different sampling depths from the same 900‐m^2^ plot. For all *N*_9_ *D*_0–10_ samples, we additionally collected a subsoil sample from 30 to 40 cm depth that was treated either separately (*N*_9_ *D*_30–40_) or combined with the topsoil samples (*N*_9_ *D*_0–10_). We recorded the coordinates of each individual sample or subplot by using tape measurements from a central spot and two crossing axes. In total, we collected 321 soil cores across the three sampling sites.

**TABLE 1:** Types of sampling designs and pooling strategies involved in the experiment. In the code for sampling design, *N* represents the number of subsamples and *D* denotes the sampling depth range (cm).

Sampling design	Basic reference	Depth (cm)	Area (m^2^)	Diameter of cores (cm)	Sample *N* for unpooled sampling	Sample *N* for pooled sampling
*N*_62_ *D*_0–5_	Tedersoo et al. (2021)	0–5	2500	5	62	62
*N*_40_ *D*_0–5A_		0–5	2500	5	40	40
*N*_40_ *D*_0–5B_		0–5	1400	5	40	40
*N*_9_ *D*_0–1_	Davison et al. (2012)	0–1	900	5	9	9
*N*_8_ *D*_0–10_	Pärtel et al. (2019)	0–10	100	5	8	8
*N*_5_ *D*_0–20_	Orgiazzi et al. (2018)	0–20	16	5	5	5
*N*_9_ *D*_0–10_	Guerra et al. (2021)	0–10	900	5	9	9
*N*_9_ *D*_30–40_		30–40	900	5	9	9
*N*_9_ *D*_0–40_		0–10 + 30–40	900	5	18	18
*N*_25_ *D*_0–7.5_	Delgado‐Baquerizo et al. (2019)	0–7.5	2500	2.5	—	25 (5 composites of 5 subsamples)
*N*_75_ *D*_0–7.5_		0–7.5	2500	2.5	—	75 (3 composites of 5 composites of 5 subsamples)
*N*_50_ *D*_0–7.5_	Maestre et al. (2012)	0–7.5	900	2.5	—	50 (10 composites of 5 subsamples)

Each sample was placed into a sterile paper bag and dried within 12 h of collection at 35°C in a drying cabinet. The dried soil samples were initially crushed by rubbing the plastic bag, and ca. 1 g of the finest particles were further homogenised using tungsten beads in a Retsch MM400 homogeniser (Retsch GmbH, Haan, Germany) at 30 Hz for 5 min. Then, 0.2 g of homogenised soil was weighed for DNA extraction. DNA extraction for all samples was performed using the MagAttract PowerSoil Pro DNA Kit (384) (Qiagen, Germany) in combination with a Qiagen KingFisher extraction robot following the manufacturer's instructions. To minimise contamination, we wore disposable gloves and cleaned instruments and work surfaces with 70% ethanol throughout all sample‐processing steps.

We focused on three sample pooling strategies for each site, including (1) unpooled strategy, (2) pooling of soil and (3) pooling of DNA. The unpooled strategy involves separate DNA extraction and analysis of individual samples as described above. For the DNA pooling strategy, 2 μL of each DNA extract from the samples collected within the same sampling design was mixed into a DNA pool. For the soil pooling strategy, ca. 3 g of the hand‐crushed samples collected within the same design were taken to the composite sample, followed by mixing, homogenisation by bead beating and DNA extraction from 0.2 g of material.

### 2.2 PCR and Sequencing

Soil samples were analysed for the animal, fungal and bacterial biodiversity based on DNA metabarcoding. We conducted PCR amplification using the universal eukaryotic primers ITS9mun (5′ GTACACACCGCCCGTCG 3′) and ITS4ngsUni (5′ CGCCTSCSCTTANTDATATGC 3′) for amplification of the fungal Internal Transcribed Spacer (ITS) marker following Tedersoo et al. (2021). The primers COI.intF (5′ GGWACWGGWTGAACWGTWTAYCCYCC 3′) and COI.2198 (5′ TAIACYTCIGGRTGICCRAARAAYCA 3′) were used for amplifying the mitochondrial Cytochrome Oxidase I (COI) gene following Anslan et al. (2021). The primers 515F (5′ GTGYCAGCMGCCGCGGTAA 3′) and 926R (5′ GGCCGYCAATTYMTTTRAGTTT 3′) were used to amplify the bacterial 16S rRNA gene as described in Bahram et al. (2018). The PCR products were visualised on a 1% agarose gel. DNA concentrations were assessed using Qubit 3.0 (Thermo Fisher Scientific, Chicago, USA).

The PCR products of the respective libraries were pooled based on the product strength on a gel, by mixing 0.5 to 20 μL of PCR product. The mixtures were shipped to a service provider for library preparation and sequencing. The animal COI and bacterial 16S regions were sequenced on an Illumina NovaSeq 6000 at the Novogene (Cambridge, United Kingdom), whereas the fungal ITS region was sequenced on a PacBio Sequel II at the Norwegian Sequencing Centre, University of Oslo (Oslo, Norway). Illumina NovaSeq 2 × 250 paired‐end protocol was used for sequencing the COI and 16S amplicons. The fungal samples producing < 2000 reads and bacterial and animal amplicons with < 50,000 reads were subjected to resequencing in another sequencing library.

### 2.3 Bioinformatics

For each group (fungi, bacteria and animals), we followed current best practices and applied state‐of‐the‐art analytical workflows. Bioinformatics analysis of sequence data of the ITS region was conducted using the NextITS pipeline v.0.5.0 (Mikryukov et al. 2025). The fungal sequences were demultiplexed with LIMA v.2.7.1 (Pacific Biosciences; https://lima.how/) using the minimum barcode score of 93. Full‐length ITS sequences were extracted using ITSx v.1.1.3 (Bengtsson‐Palme et al. 2013). Chimeric sequences were removed using a two‐step approach: de novo detection using the UCHIME algorithm (Edgar et al. 2011) with a maximum chimera score of 0.6 (Nilsson et al. 2015) and a reference‐based method using the EUKARYOME database v.1.7 (Tedersoo et al. 2024). Sequence clustering was performed using VSEARCH v.2.28.1 (Rognes et al. 2016) at a 98% sequence similarity threshold (Bradshaw et al. 2023). For post‐clustering curation, we used the LULU algorithm (Frøslev et al. 2017) with a sequence similarity threshold of 95%. To taxonomically annotate fungal and bacterial sequences, we used the Naïve Bayes classifier (Wang et al. 2007) implemented in QIIME2 v.2023.9.1 (Bolyen et al. 2019) along with the EUKARYOME reference database. Sequences were assigned to the lowest taxonomic level for which the classification bootstrap confidence was at least 80% (Bokulich et al. 2018). Non‐fungal OTUs and sequences unclassified at the kingdom level were excluded from further analyses.

The bacterial dataset was analysed using DADA2 v.2023.9.0 (Callahan et al. 2016) as implemented in the QIIME2 plugin. We removed the forward reads < 219 bp and reverse reads < 216 bp as low‐quality sequences. The reads were clustered using a 97% sequence similarity threshold (Liu et al. 2023). We used a weighted taxonomic classifier based on the SILVA database v.138.1 (Kaehler et al. 2019; Quast et al. 2013; Robeson II et al. 2021). Unclassified, non‐target (e.g., mitochondrial and plastid) and eukaryotic OTUs were excluded from the dataset. OTUs with a taxonomic confidence value < 80% at the kingdom level were removed (Bokulich et al. 2018).

For the analysis of COI amplicons, ASVs were obtained using MetaWorks v.1.13.0 (Porter and Hajibabaei 2022) with default settings. OTUs were calculated based on sequence clustering at a 99% sequence similarity threshold (Zhang and Bu 2022). Taxonomic annotation of OTUs was conducted using the RDP Classifier v.5.1.0 (Wang et al. 2007). Only OTUs assigned to Rotifera, Nematoda, Arthropoda and Annelida were retained, and those with a phylum‐level taxonomic confidence value below 80% were removed (Bokulich et al. 2018).

To understand the contribution of potential artefacts and biological species to the pooling effect, we focused on the three largest fungal groups, *Thelephoraceae*, *Sebacinaceae* and *Russulaceae*, where we had some taxonomic expertise. Representative sequences of each OTU and best‐matching reference sequences were aligned using MAFFT v.7.525 (Katoh and Standley 2013) with default options. The first step in artefact recognition was performed using Aliview v.1.30 (Larsson 2014). The putative artefacts included sequences with (i) long indels (> 20 bases throughout the sequence); and (ii) more than one single‐nucleotide indel in the 200‐base window spanning the entire 5.8S rRNA gene and first part of ITS2. The remaining sequences were subjected to Maximum Likelihood tree construction using IQ‐Tree v.2.1.4 (Minh et al. 2020) using default options. In the second step of artefact recognition, taxa with relatively long branches compared with the best hit, corresponding to > 2% sequence divergence, were removed.

Finally, we removed the samples with < 100 reads, retaining 317 individual subsamples for bacteria, 310 for fungi and 295 for animals. We retained all 36 DNA‐pooled samples for animals, bacteria and fungi, as well as 36 soil‐pooled samples for animals and bacteria and 35 soil‐pooled samples for fungi.

### 2.4 Statistical Analysis

All statistical analyses were conducted in R v.4.3.1 (R Core Team 2023). Sample completeness was estimated using Good's coverage (Chiu and Chao 2016; Kortmann et al. 2025). Coverage was near‐saturated (animals: 1.00 [median] ± 0.00 [SD], bacteria: 1.00 ± 0.0003; fungi: 0.96 ± 0.04). Therefore, to control for sequencing effort, analyses of the effects of sampling design, pooling strategy, or sampling intensity (number of samples) on community diversities were run on sequencing depth‐standardised data. For animals, bacteria and fungi separately, OTU tables were rarefied to the near‐minimum read number (animals: 880; bacteria: 1067; fungi: 814 in unpooled samples; animals: 15,962; bacteria: 39,290; fungi: 14,409 in pooled samples). To reduce rarefaction stochasticity, we performed multiple rarefactions (1000 iterations) and averaged the resulting diversity estimates. This analysis was performed using metagMisc v.0.5.0 (Mikryukov 2025) and phyloseq packages v.1.44.0 (McMurdie and Holmes 2013).

#### 2.4.1 Effect of Sampling Design and Pooling Strategy on Diversity

Rarefied OTU accumulation curves were constructed using the vegan package v.2.6‐4 (Oksanen et al. 2015). Cumulative abundance curves were built to show the OTU detection ability of each design. We compared the mean (within‐sample) OTU richness across the unpooled sampling designs using a mixed linear model, with the site as a random factor. For pooled sampling designs, the site was treated as a fixed factor. We compared the soil and DNA pooling in diversity estimates based on the linear model where site and sampling design were fixed factors. These analyses were performed using the marginaleffects package v.0.18.0 (Arel‐Bundock et al. 2024) based on the model built by the lme4 package v.1.1‐35.1 (Bates et al. 2015).

To test the effect of unpooled sampling design, we compared the obtained total richness values (within‐site) of each unpooled sampling design based on a linear model with the sampling sites and sampling designs as fixed factors using the marginaleffects and lme4 packages. We estimated sample coverage and extrapolated site‐richness using species‐by‐sampling‐site‐unit matrices as inputs based on the iNEXT.3D v.1.0.4 package (Chao et al. 2021). We regressed species richness on the sampling area using a linear model, with the site as a fixed factor, implemented in the lmerTest v.3.1‐3 package (Kuznetsova et al. 2017). Eight samples (4 pairs) were selected from the *N*_62_ *D*_0–5_ sampling design to test the sampling area effect on richness.

We performed the permutational multivariate analysis of variance (PERMANOVA; Anderson 2017) to compare community composition across sampling designs based on the Bray‐Curtis distances (vegan package) among individual samples. To assess the effect of sampling design on beta‐diversity estimates, we conducted the homogeneity test of multivariate dispersion at each site using the Bray‐Curtis distance using the vegan package. To test the impact of the sample pooling on community composition similarity among sampling designs, we performed the marginal contrast with site as a random factor and designs as a fixed factor based on Bray‐Curtis distance using vegan, marginaleffects, and lme4 packages.

#### 2.4.2 Pooling Effect Analysis

We define the pooling effect as the ratio of OTU richness or effective number of OTUs (Jost 2006) between pooled and unpooled sampling obtained with the same sampling design. Although the Shannon index is widely used, its logarithmic basis makes interpreting its ratios unintuitive. Exponentiating the Shannon index yields an effective number of OTUs, corresponding to Hill number of *q* = 1 and representing the number of equally abundant species required to achieve the observed diversity and facilitating more intuitive comparisons among sampling designs. To compare the pooling effects among various sampling designs, the summary sequencing depth of each sampling design (the total reads of the same unpooled design) was used to assess the pooling effect within each design (Table S1). We compared the effects of pooling strategy (soil vs. DNA) using a linear mixed‐effects model, with sampling site and sequencing depth as random effects, sampling design and pooling strategy as fixed effects. The analyses were conducted with the lme4 and marginaleffects packages. The effect sizes of the fixed effects were estimated as the partial eta‐squared ($\eta_{p}^{2}$) using the effectsize v.0.8.6 package (Ben‐Shachar et al. 2020).

To assess the artefacts and rare species attribution to the pooling effect, we compared the proportion of artefact (*N*(artefacts)/*N*(OTUs)), rare OTUs (*N*(rare)/*N*(OTUs)) and rare artefact (*N*(artefacts)/*N*(rare species)) and the number of unique OTUs, rare artefacts, and unique artefacts in *Thelephoraceae*, *Sebacinaceae* and *Russulaceae*. The comparison was conducted using a mixed linear model with sampling designs, and the pooling strategy as fixed factors and the sampling site as a random factor based on the lme4 and the marginaleffects package. Model parameters and performance were assessed using the parameters v.0.21.6 and performance v.0.11.0 packages (Lüdecke et al. 2020, 2021). Species with relative abundance ≤ 0.05% of total reads were defined as rare species.

We evaluated the extent of resource savings achieved through the pooling strategies using calculator available at https://mycology‐microbiology‐center.github.io/Soil_biodiversity_assessments/. In this work, the pooling effect for DNA and soil pools was evaluated at varying sequencing depths, reflecting the proportions of the expenses for sequencing a pooled sample relative to the sequencing of all unpooled samples within the same design. This analysis highlights how pooling strategies can reduce resource consumption while maintaining effective community composition and diversity assessments. The costs are measured in working hours and units of chemicals used for DNA extraction, PCR and sequencing. One working hour corresponds to roughly 10 extracted DNA samples or 40 PCR reactions (including product check and purification). We used a linear model to analyse the pooling strategy's impact on the pooling effect, where the site, sequencing depth and sampling designs were treated as fixed factors using the lme4 and marginaleffects packages.

## 3 Results

### 3.1 Individual Sample Diversity Varies Across Sampling Designs

**FIGURE 2:**
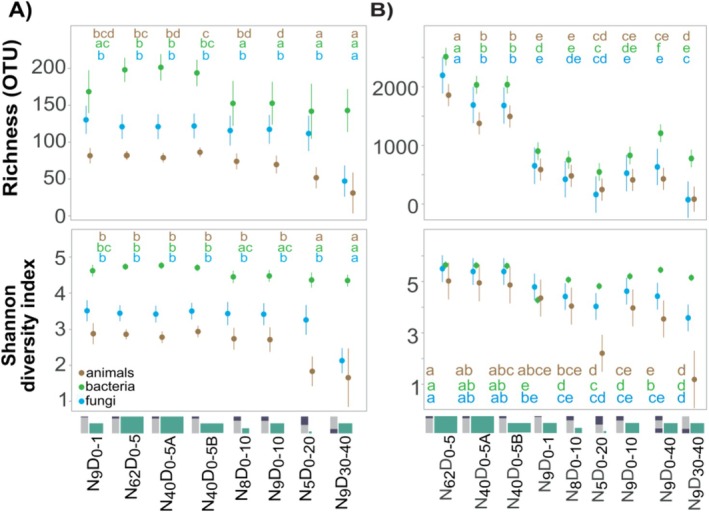
Diversity of soil organisms across sampling designs in individual samples. (A) Marginal estimates of mean OTU richness and the Shannon diversity index in individual samples across sampling designs. (B) Marginal estimates of mean within‐site OTU richness and Shannon diversity index. Similar letters group sampling designs with mean values that do not differ significantly (*α* = 0.05).

Diversity estimates in individual soil samples differed across sampling designs, depending on sampling depth and target taxon (Figure 2A, Table S2). The highest animal OTU richness was obtained with the *N*_9_ *D*_0–1_ and *N*_62_ *D*_0–5_ designs, with a gradual decrease through the *N*_8_ *D*_0–10_ and *N*_9_ *D*_0–10_ design to *N*_5_ *D*_0–20_ and *N*_9_ *D*_30–40_ designs. Bacterial OTU richness was significantly higher in the *N*_62_ *D*_0–5_ and *N*_40_ *D*_0–5A_ designs compared with all the other designs. The bacterial Shannon diversity index gradually decreased from the designs sampling within 0–5 cm through the designs sampling within 0–10 cm to *N*_5_ *D*_0–20_ and *N*_9_ *D*_30–40_. Conversely, fungal OTU richness did not significantly differ among sampling designs, except for much lower values in the *N*_9_ *D*_30–40_ design.

Based on PERMANOVA tests, community composition dissimilarity among sampling designs was slightly but significantly greater than within the designs (animals: *F*_(7,449)_ = 1.3, *R* ^2^ = 0.02, *p* = 0.002; bacteria: *F*_(7,531)_ = 3.4, *R* ^2^ = 0.04, *p* = 0.001; fungi: *F*_(7,459)_ = 1.5, *R* ^2^ = 0.02, *p* = 0.001; Tables S3 and S4). Animal communities of the *N*_5_ *D*_0–20_ samples significantly differed from those collected from the uppermost topsoil (*N*_9_ *D*_0–1_ and *N*_62_ *D*_0–5_ designs); however, no significant differences were observed aside from these. Bacterial communities of 0–1 cm, 0–5 cm, 0–20 cm and 30–40 cm depth all differed from each other but (except for *N*_9_ *D*_30–40_ and *N*_9_ *D*_0–1_) overlapped with the designs assessing 0–10 cm depth. In fungi, samples obtained from 0–1 cm, 0–5 cm, 0–10 cm and 0–20 cm depth had generally similar fungal communities, except for significant differences in the 0–1 cm topsoil compared with 0–20 cm. Subsoil fungal communities of *N*_9_ *D*_30–40_ samples differed from other sampling designs, except the *N*_5_ *D*_0–20_ design (Table S3).

Sampling designs significantly differed in the variability of community composition (Table S4), with the lowest dispersion in *N*_5_ *D*_0–20_ (animals: 0.236, bacteria: 0.339, fungi: 0.326). The *N*_62_ *D*_0–5_ (0.670), *N*_9_ *D*_30–40_ (0.447), and *N*_62_ *D*_0–5_ (0.597) showed the highest dispersion in the composition of animals, bacteria, and fungi, respectively.

### 3.2 Sampling Design Influences Overall Biodiversity Estimates

The total OTU richness in the site, assessed based on unpooled sampling, was the highest for the topsoil sampling designs (depth: 0–5) with a high number of subsamples (Figure 2B, see Table S2). The richness ratios between sampling designs reached 26.8, 5.9 and 14.7 for animals, bacteria and fungi, respectively. Specifically, the *N*_40_ *D*_0–5_ design yielded, respectively, 2.4–22.1, 1.7–4.8 and 1.7–11.5 times more animal, bacterial and fungal OTUs than designs with fewer subsamples (subsoil *N*_9_ *D*_30–40_ was excluded from ratio calculation). The *N*_5_ *D*_0–20_ and *N*_9_ *D*_30–40_ designs generally revealed significantly lower numbers of OTUs per site compared to the other designs.

Compared to the *N*_9_ *D*_0–10_ design, the *N*_9_ *D*_0–40_ design (i.e., merging topsoil and subsoil samples) added on average 4.8%, 54.1% and 17.3% to OTU richness of animals, bacteria and fungi, respectively; yet these values were significantly below the values of *N*_62_ *D*_0–5_ and *N*_9_ *D*_0–1_ designs, suggesting that an extra effort of sampling subsoil or deep cores does not add much to the recovered richness, except in bacteria. Across all sites, subsoils harboured only 0.6%, 4.9% and 1.5% of unique OTUs of animals, bacteria and fungi, respectively.

In all organism groups, the Shannon diversity index varied similarly to richness, decreasing from the topsoil samples (depth: 0–5 cm) through the *N*_9_ *D*_0–1_, *N*_9_ *D*_0–10_ and *N*_8_ *D*_0–10_ designs to the *N*_5_ *D*_0–20_ and *N*_9_ *D*_30–40_ designs (Figure 2B, see Table S2). The ratios of effective numbers of OTUs between *N*_40_ *D*_0–5_ and other designs with fewer subsamples (excluding *N*_9_ *D*_30–40_) were 1.1–60 for animals, 1.1–4.7 for bacteria and 1–6.9 for fungi. For all groups of organisms, the topsoil samples detected more low‐abundance OTUs and a lower proportion of dominant OTUs than those from the other designs (Figures S1 and S2). The *N*_5_ *D*_0–20_ and *N*_9_ *D*_30–40_ unpooled sampling designs captured the fewest low‐abundance OTUs.

**FIGURE 3:**
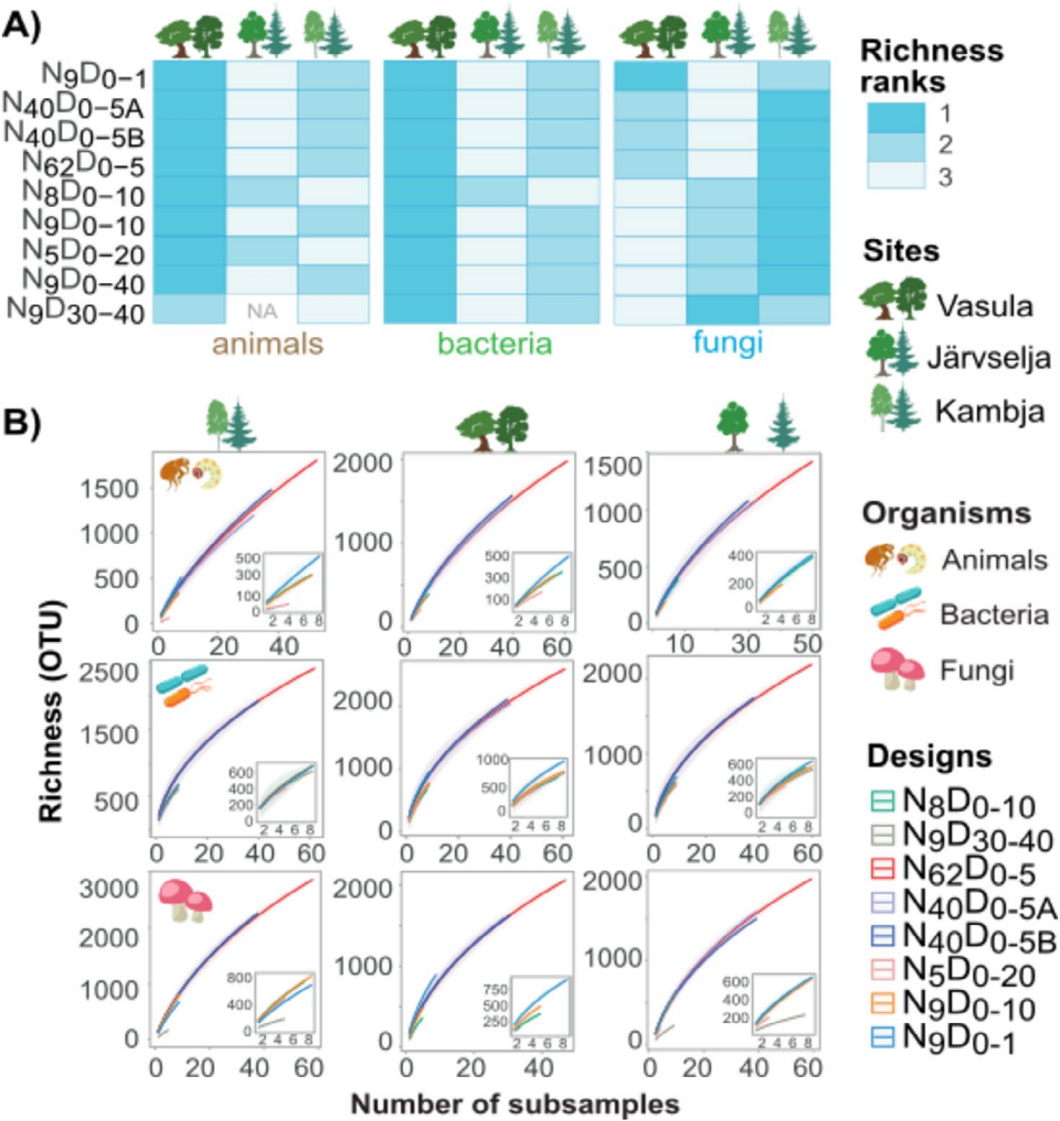
Relative performance of sampling designs in analysing within‐site diversity. (A) Within‐site richness ranked within each sampling design. (B) OTU accumulation curves with sampling effort for different sampling designs.

We further analysed if sampling design affects the patterns of diversity distribution across sampling sites (Figure 3A). For animal richness, site ranking performed with *N*_5_ *D*_0–20_ and *N*_8_ *D*_0–10_ deviated from the other designs (Figure 3A). For fungi, the designs involving 0–5 cm depth ranked the sites in the order Kambja‐Vasula‐Järvselja, while those sampling within 0–10 and 0–20 cm ranked in the order Kambja‐Järvselja‐Vasula. Designs sampling from the 0–1 cm and 30–40 cm depth deviated from both above patterns in fungi. In bacterial richness estimates, all sampling designs, except *N*_8_ *D*_0–10_, ranked sites identically.

For most microbial groups, the rate of OTU accumulation with sampling effort was the greatest for the designs focusing on the upper soil layers (Figure 3B). Sampling only the subsoil (*N*_9_ *D*_30–40_ design) yielded the lowest rate of OTU gain for animals and fungi, while bacterial richness accumulation was slowest in the *N*_5_ *D*_0–20_ design.

We specifically tested the importance of sampling area on richness recovery by subsampling 8‐sample combinations (*N*(animal) = 35; *N*(bacteria) = 47; *N*(fungi) = 31) within the *N*_62_ *D*_0–5_ design while varying the rectangular (|width—length| ≤ 5 m) sampling area from 327 to 2000 m^2^. The sampling area had no effect on OTU richness in any of the organism groups (*p* > 0.05 in all cases; Table S5). Based on all topsoil samples, animals, bacteria, and fungi exhibited a significant positive spatial autocorrelation in community composition (Figure S3).

### 3.3 Sample Pooling Reduces Differences Among Sampling Designs

**FIGURE 4:**
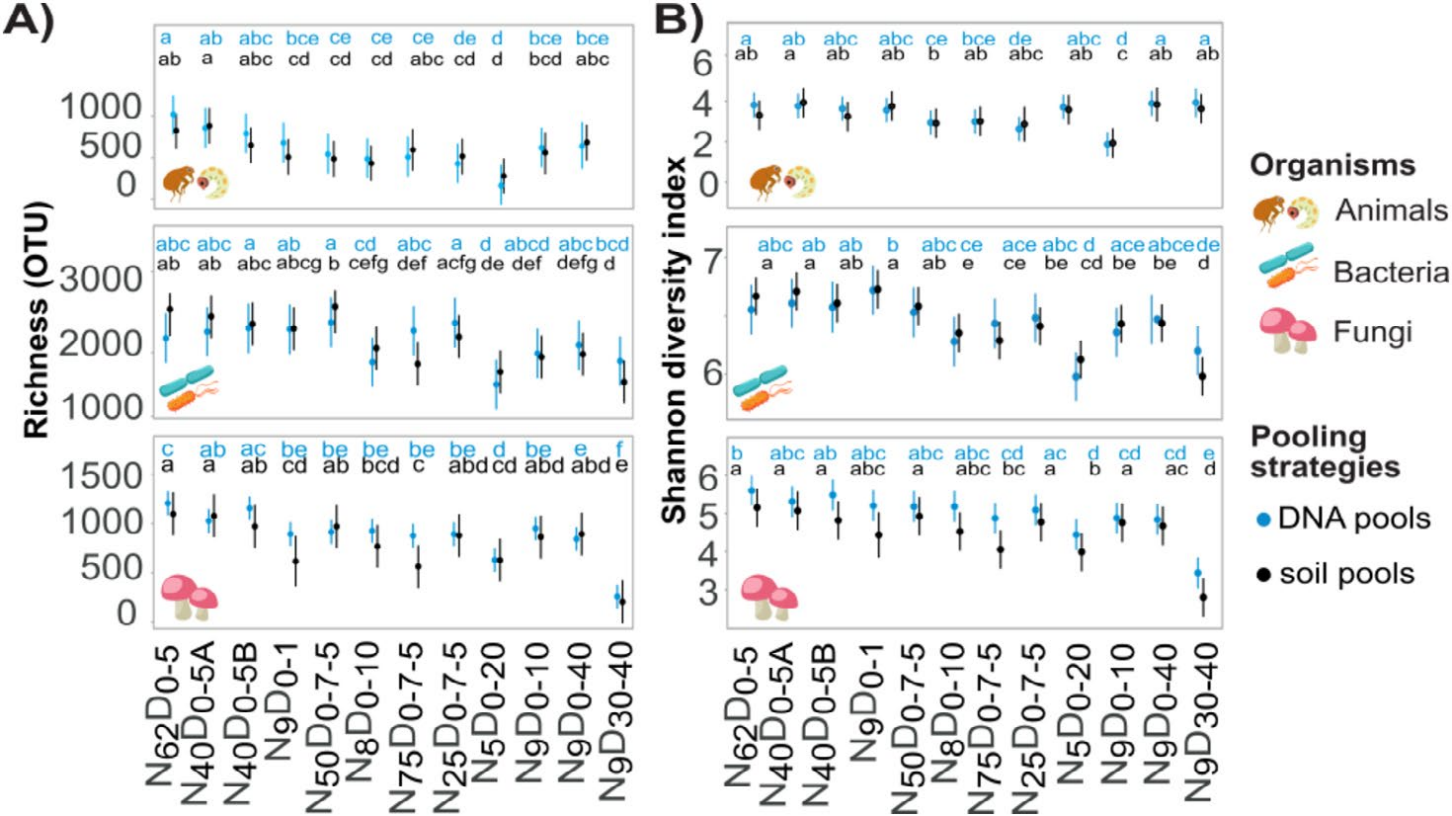
Within‐site diversity captured by sample pooling designs. (A) Marginal means for OTU richness captured with soil and DNA pools across sampling designs in animal, bacterial and fungal communities. (B) Marginal means for the Shannon diversity index. Linear models were used, with sampling sites and sampling designs treated as fixed factors. Similar letters mark sampling designs with statistically (*α* = 0.05) similar mean values.

To compare the sampling designs in diversity estimates obtained with pooled samples, we rarefied the samples at near‐minimal sequencing depths, that is, at 15,962, 39,290 and 14,409 sequences for animals, bacteria and fungi, respectively. Similarly to unpooled samples, pooled samples of the *N*_62_ *D*_0–5_ design revealed the highest numbers of OTUs and the highest Shannon index (Figure 4A,B, Table S6) due to a greater number of low‐abundance OTUs (see Figures S1 and S2). However, the variability of richness and Shannon diversity index across pooling sampling designs was lower than across sampling designs with unpooled sampling. Specifically, in pooled samples, the richness ratio between *N*_40_ *D*_0–5_ and other designs (excluding *N*_9_ *D*_30–40_) were 0.9–3.1, 0.8–2.2 and 0.9–3.5 folds for animals, bacteria and fungi, respectively. The ratios of effective numbers of OTUs were 0.3–20, 0.8–2.4 and 0.7–5.8 folds for animals, bacteria and fungi, respectively. Compositional similarity between sampling designs was higher with pooled sampling than with unpooled sampling for bacteria (marginal contrast MC_UNPOOLED‐SOIL_ = −0.05, *p* = 0.047; MC_UNPOOLED‐DNA_ = −0.06, *p* = 0.005; see Table S5) and fungi (MC_UNPOOLED‐SOIL_ = −0.08, *p* < 0.01; MC_UNPOOLED‐DNA_ = −0.11, *p* < 0.001) but not for animals (MC_UNPOOLED‐SOIL_ = 0.04, *p* = 0.2; MC_UNPOOLED‐DNA_ = −0.04, *p* = 0.2).

In contrast to unpooled sampling, increasing the pooled subsamples from 40 to 62 did not significantly increase OTU richness. The lowest OTU richness in both DNA and soil pools was revealed with *N*_9_ *D*_30–40_ and *N*_5_ *D*_0–20_ designs. *N*_50_ *D*_0–7.5_, *N*_25_ *D*_0–7.5_ and *N*_75_ *D*_0–7.5_ designs generally captured fewer OTUs than *N*_40_ *D*_0–5_ designs in animals (up to 1.8‐fold) and fungi (up to 6‐fold). Meanwhile, the *N*_50_ *D*_0–7.5_ design revealed slightly higher bacterial richness than other designs. Across all designs, the DNA pooling strategy revealed more animal and fungal OTUs than the soil pooling strategy (MC_DNA‐SOIL_ = 147.1, *p* = 0.01; MC_DNA‐SOIL_ = 91.4, *p* = 0.01; see Table S5), but there was no effect in bacteria (MC_DNA‐SOIL_ = −113.4, *p* = 0.2).

### 3.4 Effect of Sample Pooling Across Different Sequencing Depths

**FIGURE 5:**
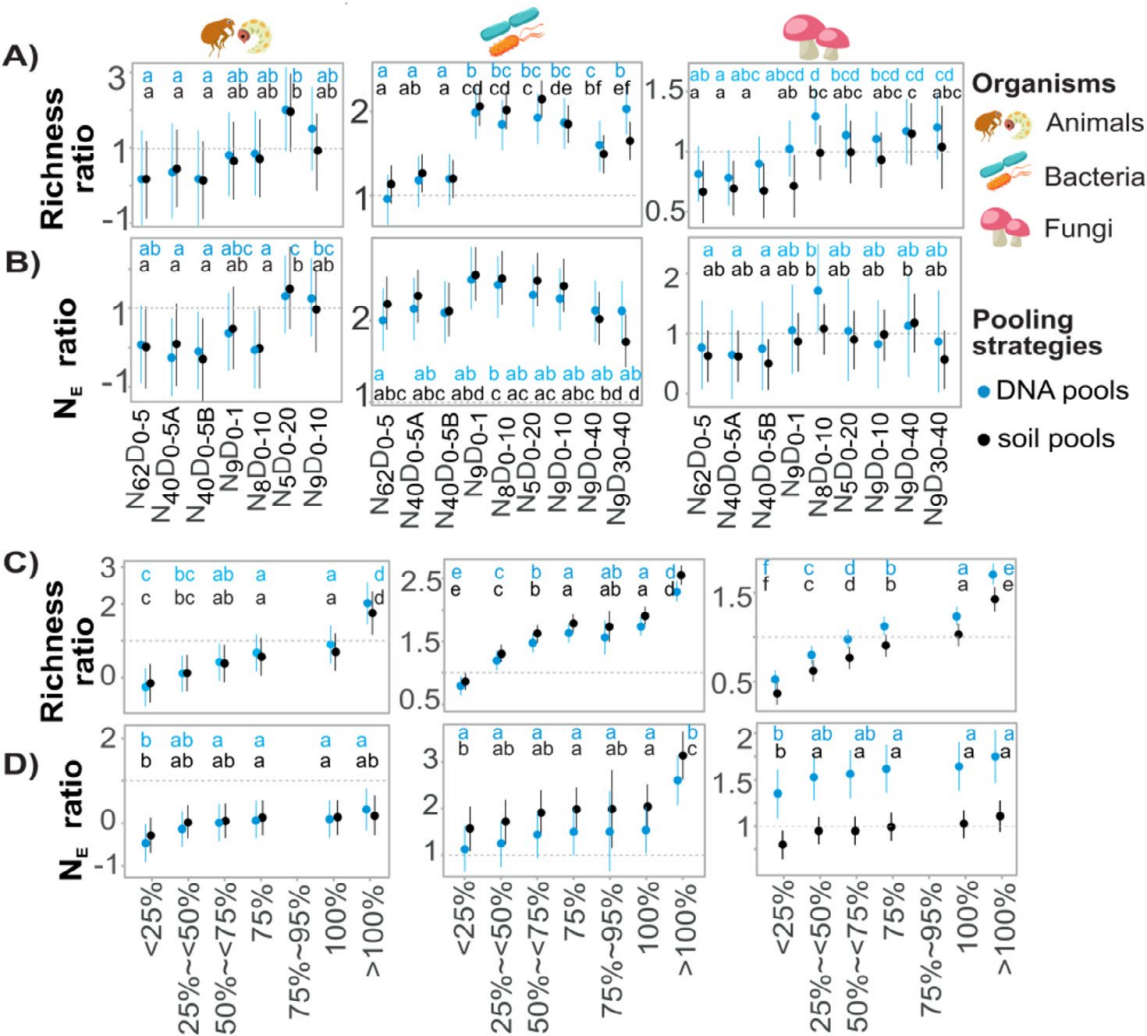
Pooling effect across sequencing depths and sampling designs. Pooling effect (ratio of within‐site diversity estimates between pooled and unpooled samples) for (A) OTU richness and (B) effective number of OTUs across different sampling designs at the 100% summary sequencing depth. Pooling effect on (C) richness estimates and (D) effective number of OTUs (*N*_E_) across sequencing depths. The marginal means were derived from linear models, where sampling sites and sampling designs are treated as fixed factors. The x‐axis represents the percentage of reads in pooled samples from the summary number of reads in unpooled samples within the same sampling design. Similar letters denote sampling designs with statistically (*α* = 0.05) similar mean values.

To understand the pooling effect (PE) on diversity estimates across various sampling designs, we rarefied the pooled samples at the total sequencing depth of unpooled samples within the same design (i.e., 100% summary sequencing depth; Table S1) and defined PE as the ratio of OTU number between pooled and unpooled samples. It shows the gain (PE > 1) or loss (PE < 1) in OTU numbers captured with pooled compared to unpooled sampling. The range of PE_100%_ was 0.4–5.8, 0.5–2.4 and 0.6–1.7 in animals, bacteria and fungi, respectively, depending on sampling design and pooling strategy (Figure 5A,B, Table S7). For the densely sampled *N*_62_ *D*_0–5_, *N*_40_ *D*_0–5A_ and *N*_40_ *D*_0–5B_ designs, sample pooling caused OTU loss (PE_100%_ < 1) in animals and fungi. In contrast, DNA and only *N*_9_ *D*_30–40_ soil pooling design enhanced fungal OTU recovery in other sampling designs (PE_100%_ > 1) in fungal communities. Soil and DNA pooling enhanced bacterial OTU recovery in all sampling designs, except for *N*_62_ *D*_0–5_. Additionally, DNA and soil pooling only improved the ability of *N*_5_ *D*_0–20_ and *N*_9_ *D*_0–10_ to capture animal OTUs.

The PE varied with sequencing depth (Figure 5C,D, see Table S7), differing among groups of organisms. The DNA and soil pooling did not improve animal diversity detection (PE < 1) at ≤ 100% summary sequencing depth. However, respectively, for bacteria and fungi, roughly 25% and 75% sequencing effort is enough to outperform unpooled sampling in OTU recovery.

The PE on richness was relatively higher in DNA pools than in soil pools for animals and fungi (animals: MC_SOIL‐DNA_ = −0.2, *p* = 0.03; fungi: MC_SOIL‐DNA_ = −0.08, *p* < 0.01; see Table S5), while soil pooling revealed relatively more bacterial OTUs (MC_SOIL‐DNA_ = 0.08, *p* = 0.006). Based on the PE calculated for Shannon diversity indices, soil pooling generally retrieved more low‐abundance fungal and bacterial OTUs than DNA pools, but there were no differences in animals.

Based on analyses performed for this study, we calculated that for the *N*_40_ *D*_0–5_ design, sample pooling at the sampling stage (i.e., soil pooling) for 100 plots (4000 individual samples) saves 97.3% of resources destined for unpooled samples for chemicals, 76.9% of cost for sequencing library preparation plus 365 person‐hours (97.3%) in the analysis of all animals, fungi and bacteria at the similar sequencing depth. For *N*_9_ *D*_0–10_ and *N*_5_ *D*_0–20_ designs, savings for labour are 74.7 (87.9%) and 37.4 (78.4%) person‐hours, respectively, and savings for chemicals are 88.9% and 80%, respectively. Savings for sequencing are the same.

### 3.5 Contribution of Molecular Analysis Artefacts in Pooling Effect

To understand the mechanisms of sample pooling effect on biodiversity estimates, we assessed whether PE could be attributable to rare species or undiscovered molecular analysis artefacts. We compared unpooled and pooled samples in the numbers and proportions of unique (detected in a site by only one design), rare (≤ 0.05% total reads), and artefactual OTUs at 100% summary sequencing depth. DNA pools, especially those with PE < 1, detected significantly fewer unique (up to 4.9 times) and rare (up to 1.47 times) OTUs and produced fewer unique artefacts (up to 11 times) compared to unpooled samples (Table 2, Table S8). These patterns were especially pronounced in the Vasula sampling site. Soil pools did not differ in the numbers of rare, unique, and artefactual OTUs from unpooled samples. Meanwhile, both DNA and soil pooling captured a smaller number of rare artefacts (up to 2 and 1.67 times for DNA and soil pools, respectively) but resulted in a higher proportion of rare artefacts (up to 1.6 times).

**TABLE 2:** Marginal contrasts (MC) in the proportion and counts of fungal rare, unique and artefactual OTUs between unpooled samples and DNA or soil pools.

Parameters	Unpooled‐DNA					Unpooled‐soil				
	MC	*p*	*Z* value	*R* ^2^	$\eta_{p}^{2}$	MC	*p*	*Z* value	*R* ^2^	$\eta_{p}^{2}$
Unique OTUs (PE > 1)	5.1	0.07	1.8	0.33	0.1	−0.2	0.9	−0.1	0.4	0.001
Unique OTUs (PE < 1)	**18.4**	**< 0.001**	**6.4**	**0.8**	**0.7**	6.5	0.07	1.8	0.4	0.1
Unique OTUs	**9.5**	**< 0.001**	**2.5**	**0.5**	**0.3**	4.2	0.1	1.6	0.5	0.1
Rare OTUs (PE > 1)	0.6	0.7	0.5	0.7	0.01	0.4	0.8	0.3	0.7	0.01
Rare OTUs (PE < 1)	**9.8**	**0.01**	**2.5**	**0.9**	**0.2**	1.9	0.6	0.5	0.9	0.2
Rare OTUs/all OTUs	0.01	0.8	0.3	0.4	0.01	0.02	0.4	0.9	0.4	0.01
All artefacts/all OTUs	0.02	0.4	0.9	0.5	0.03	**−**0.01	0.6	−0.5	0.5	0.03
Unique artefacts	**3.1**	**< 0.01**	**3**	**0.5**	**0.2**	0.5	0.7	0.4	0.5	0.004
Rare artefacts	1.8	0.3	0.3	0.7	0.02	1.4	0.4	0.8	0.7	0.02
Rare artefacts/rare OTUs	**−0.2**	**< 0.001**	**−3.6**	**0.3**	**0.2**	**−0.1**	**0.01**	**−2.5**	**0.3**	**0.2**

*Note:* Bold values indicate statistically significant differences between groups at *α* = 0.05.

## 4 Discussion

### 4.1 Sampling Designs Differ in Diversity Estimates

We showed that with unpooled sampling, the differences between sampling designs in richness estimates may exceed 27‐fold. This depends on the taxonomic group of organisms and properties of the sampling design, including soil depth, number of subsamples, and sampling area. Our analyses show that species of animals, bacteria, and fungi continue accumulating with an increasing sampling effort of more than 200 individual samples, indicating that sampling effort contributes substantially to the differences in recovered diversity among sampling designs. In agreement, the sampling designs *N*_50_ *D*_0–7.5_ and *N*_75_ *D*_0–7.5_ recovered the highest number of species. Our extrapolation shows that to reach half the richness of a deeply sampled community (200 individual samples per site), 56–62, 48–53, and 43–49 samples would be needed for animals, bacteria, and fungi from our study sites. We recommend at least 20 individual samples (see also Tedersoo et al. 2022), because designs with < 10 samples were inconsistent in ranking the sites by richness.

Typically, the number of individual samples and sampling area are correlated (Dickie et al. 2018). Here, we specifically tested the importance of sampling area (i.e., plot size) on richness recovery based on subsampling the *N*_62_ *D*_0–5_ design. Within the range of 327–2000 m^2^, the size of the sampling area did not affect the overall richness of soil biota. Similarly, reducing the sampling area from 2500 to 1400 m^2^ using 40 subsamples resulted in comparable richness estimates. However, our layout of individual samples does not allow evaluation of whether the low diversity revealed with the *N*_5_ *D*_0–20_ design is primarily due to an insufficient number of subsamples (*N* = 5) or to its exceptionally small area (16 m^2^) or the dilution effect of mineral soil below 5–10 cm. We recommend selecting an area large enough to enable collecting a sufficient number of samples (at least 20) outside the spatial autocorrelation range, that is, a minimum area of 347, 428 and 645 m^2^ for 8 animal, bacterial and fungal individual samples, respectively.

Unlike Beule et al. (2022) and Eilers et al. (2012), we found that sampling within the top 5 cm or 10 cm is preferable for characterising the greatest part of the local soil biota. More deeply sampled soil columns harboured fewer species of fungi and bacteria compared with shallower ones. Subsoil samples harboured lower richness of all organism groups, corresponding to the previously observed vertical stratification of microbial diversity (Allison et al. 2007; Fierer et al. 2003; Queiroz et al. 2015; Schlatter et al. 2018). Furthermore, subsoil samples harboured a low proportion of unique OTUs, suggesting impoverished diversity, especially for fungi and animals. Because of the potential dilution effect (Du et al. 2017), adding mineral soil below 5 cm or 10 cm to molecular analysis may produce lower richness estimates. Nonetheless, OTU composition in relatively deep and shallow samples differs (Blume et al. 2002; Schlatter et al. 2018), but whether this can be mainly ascribed to the nestedness or turnover effects (Baselga 2010; Martiny et al. 2011; Schlatter et al. 2018) remains unclear. The significant sampling depth effect indicates that the depth of sampling is a confounding parameter when comparing communities revealed using different sampling designs. Importantly, deeper sampling may reveal physicochemical properties that do not correspond to the sampled biota due to higher bulk density and lower microbial biomass of mineral soil.

### 4.2 Benefits and Limitations of Sample Pooling

The effect of sample pooling on richness estimate accuracy differs among studies, depending on the sampling effort, ecosystem type, pooling strategy, and taxonomic resolution of molecular methods (Adamo et al. 2021; Bullington et al. 2021; Engel et al. 2012; Grundmann and Gourbière 1999; Manter et al. 2010; Ranjard et al. 2003). In our research, the pooling effect (the ratio between the pooled sample and individual samples in the captured site diversity) also differs among sampling designs, organisms of interest, and sequencing depth. Specifically, the richness of fungi and especially animals was reduced by pooling, consistent with the results of Engel et al. (2012) and Manter et al. (2010). Meanwhile, in contrast to Manter et al. (2010) which found locally abundant bacterial species becoming undetectable in sample pools due to dilution, the richness of bacteria increased with pooling in our work.

We revealed that the pooling effect depends on the sampling design, particularly on the number of composed subsamples. Mixtures composed of > 20 subsamples resulted in a loss of fungal and animal (but not bacterial) richness compared to unpooled sampling (i.e., PE < 1), irrespective of sequencing depth. Conversely, pooling < 20 subsamples typically showed a gain in richness (PE > 1). As each individual sample is expected to include unique biological species because of their patchy distribution (O'Dwyer and Green 2010; Ranjard and Richaume 2001), we suggest that the loss of such unique and/or rare OTUs by sample pooling may contribute to the observed pooling effect. However, an in‐depth analysis revealed a lower number of unique artefacts in DNA‐pooled samples (but not soil‐pooled samples) than in individual samples taken together. Because each amplicon may yield specific artefactual OTUs (Acinas et al. 2005), the sum of multiple individual amplicons likely contains a larger number of unique artefacts. It is possible that increasing chimera formation in diverse pooled samples (Omelina et al. 2019) is outweighed by summing up of artefacts in individual samples. Previous studies (Dopheide et al. 2019; Epp et al. 2012; Vesty et al. 2017) could not specifically address the remaining artefacts.

Although both DNA and soil pooling retained the imprint of sample size and soil depth on richness estimates, pooling resulted in an increase in bacterial and fungal community similarity among sampling designs. In addition, the differences among sampling designs in OTU richness or the effective number of OTUs were approximately threefold smaller compared with unpooled sampling. The amplification process may favour certain species and disfavour others through PCR bias (Acinas et al. 2005). Via a sampling effect, certain species in pooled samples are preferably amplified and sequenced, which may artificially homogenise communities in pooled samples.

The main criticism of pooling is the loss of information about within‐site beta diversity and its local‐scale drivers (Manter et al. 2010; Osborne et al. 2011). Using only pooled samples, we could not have estimated, for example, the spatial autocorrelation range, sampling area effect, and optimal sample numbers. Our results also point to the homogenising effects of pooling, suggesting that biological differences among sites and ecological treatments may be more easily captured when using unpooled samples and combining sequencing data at a later stage. However, using pooled samples is feasible when comparing the data against other studies with different sampling designs due to the homogenisation effect.

The main advantage of sample pooling is the reduction of labour and analytical costs, particularly when assessing diversity across a large number of study sites (Baker et al. 2009; Osborne et al. 2011). For this study, the soil pooling saved up to 98.3% of resources, reducing labour and chemical costs 4.9‐ to 60.1‐fold. Pooling affected DNA sequencing costs much less, because pooled samples may require deeper sequencing to reach PE > 1 (i.e., comprehensive sequencing depth). In spite of minor increases in richness, DNA pooling does not offer any advantage over soil pooling, because similar homogenising effects are evident, and DNA pooling has enormous additional costs of surplus DNA extraction. Indirectly, our analyses showed that lumping up to 62 DNA extracts of 0.2 g of soil does not improve richness recovery for animals, bacteria or fungi when estimated based on a single DNA extraction from 0.2 g of homogenised soil mixture. We admit that these inferences can be restricted to the sampled sites and encourage further data accumulation in other biomes using the sampling designs involved in this study as they base global‐ and continental‐scale soil biodiversity projects (Tedersoo et al. 2021; Davison et al. 2012; Pärtel et al. 2019; Orgiazzi et al. 2018; Guerra et al. 2021; Delgado‐Baquerizo et al. 2019; Maestre et al. 2012) that underlie the largest datasets (Delgado‐Baquerizo et al. 2018; Mikryukov et al. 2023; Van Nuland et al. 2025) that are used in numerous studies on fungal and bacterial diversity and biogeography.

## 5 Conclusions

For molecular biodiversity surveys of soil, representative sampling is necessary. Given the accumulation of soil biomass and biodiversity in the top few centimetres, sampling at a depth of 5 cm is sufficient to capture a large proportion of all animal, bacterial and fungal diversity. We recommend collecting at least 30 subsamples for animals, bacteria and fungi. Additional sampling effort depends on the intended sampling area, ease of vegetation penetration and heterogeneity of studied systems. The size of the sampling area had no effect on richness estimates at 327–2500 m^2^, but it should consider spatial autocorrelation range, potential edge effects and type of vegetation (i.e., trees vs. herbs). Considering labour and analytical costs, sample pooling in situ is feasible unless local‐scale beta diversity or species co‐occurrence analyses are intended. Considering the small but significant drawbacks of pooling on diversity, 2–3 smaller soil pools (< 10 samples each) could be analysed separately, and the results summarised if the budget permits. Using pooled samples is also feasible when comparing the data against other studies with different sampling designs due to the homogenising effects.

## Author Contributions

M.C. performed the analyses, visualised and interpreted the results and wrote the manuscript. V.M. provided analytical tools. O.D. visualised the results. O.C. provided animal data. L.T. designed the research, performed sampling and provided bacterial and fungal data. M.M., V.M., O.D. and M.C. built the interface. V.M., O.D. and L.T. supervised data analysis, participated in manuscript writing and results interpretation. All authors reviewed and edited the manuscript.

## Funding

This work was supported by Estonian Research Council (PRG632) and China Scholarship Council.

## Conflicts of Interest

The authors declare no conflicts of interest.

## Supporting information


**Appendix S1:** men70113‐sup‐0001‐AppendixS1.docx.

**Appendix S2:** men70113‐sup‐0002‐AppendixS2.xlsx.

## Data Availability

Demultiplexed Illumina and PacBio reads for each sample were deposited in the European Nucleotide Archive (ENA) under the accession number PRJEB88999. The calculator for sample processing costs is available at: https://mycology‐microbiology‐center.github.io/Soil_biodiversity_assessments/. All scripts used in the statistical analysis are available under the MIT licence at GitHub: https://github.com/Mycology‐Microbiology‐Center/Soil_biodiversity_assessments.
