# Supplementary material for: Sampling Design and Sample Processing Affect Soil Biodiversity Assessments
Source: Mol Ecol Resour. 2026 Feb 24;26(3):e70113. doi: 10.1111/1755-0998.70113 (PMC12931581; doi:10.1111/1755-0998.70113)
Supplement: Supplementary file 1 — Appendix S1: men70113‐sup‐0001‐AppendixS1.docx. [file MEN-26-e70113-s002.docx]

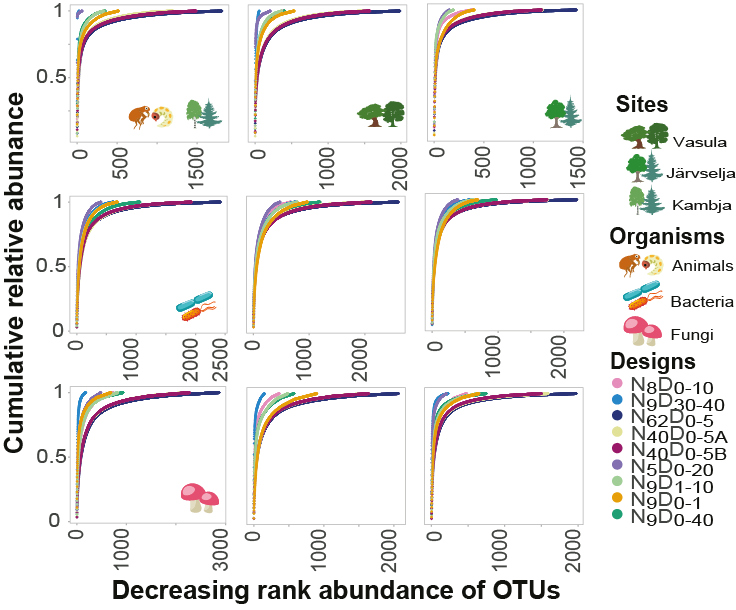


**Figure S1.** **Within-site diversity across unpooled sampling designs.** The relative cumulative abundance curves in animal, bacterial, and fungal communities. OTU numbers were ordered by decreasing relative abundance within each sampling design.


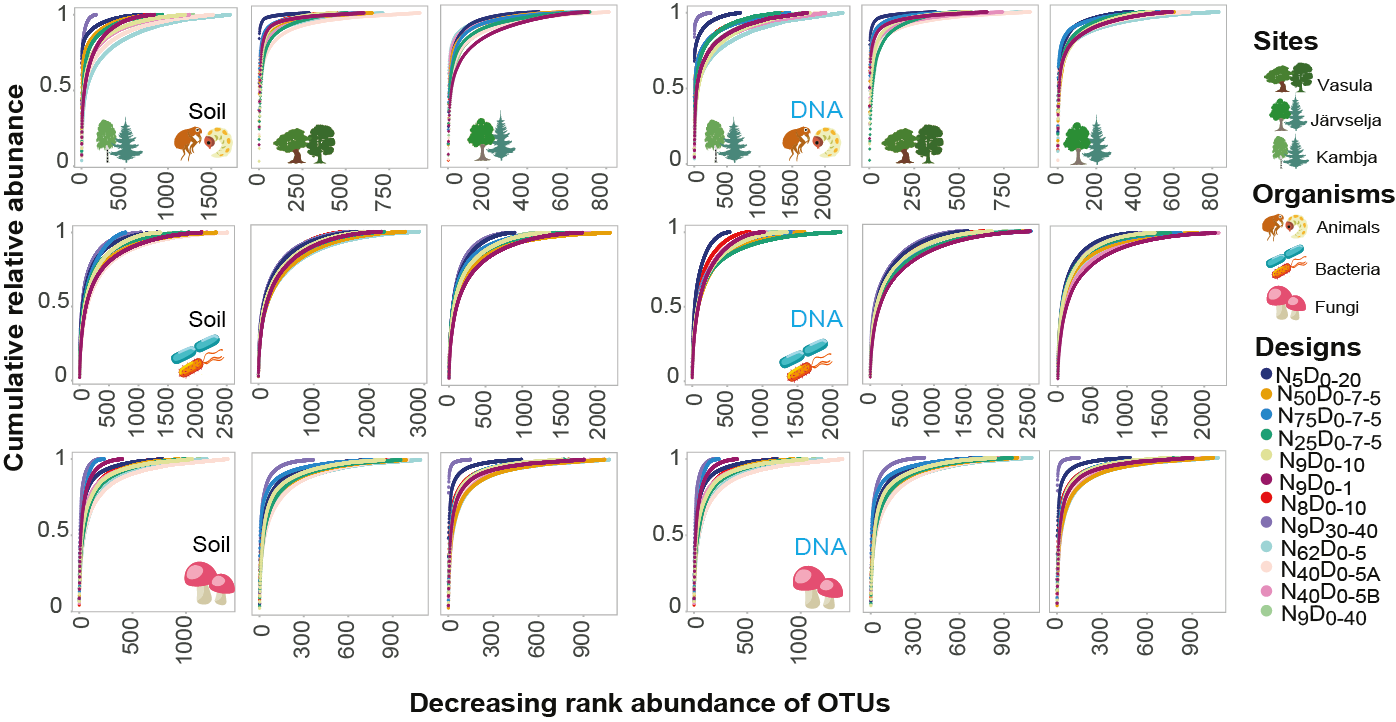


**Figure S2.** **Within-site diversity across pooling sampling designs.** The relative cumulative abundance curves in animal, bacterial, and fungal communities. OTU numbers were ordered by decreasing relative abundance within each sampling design.


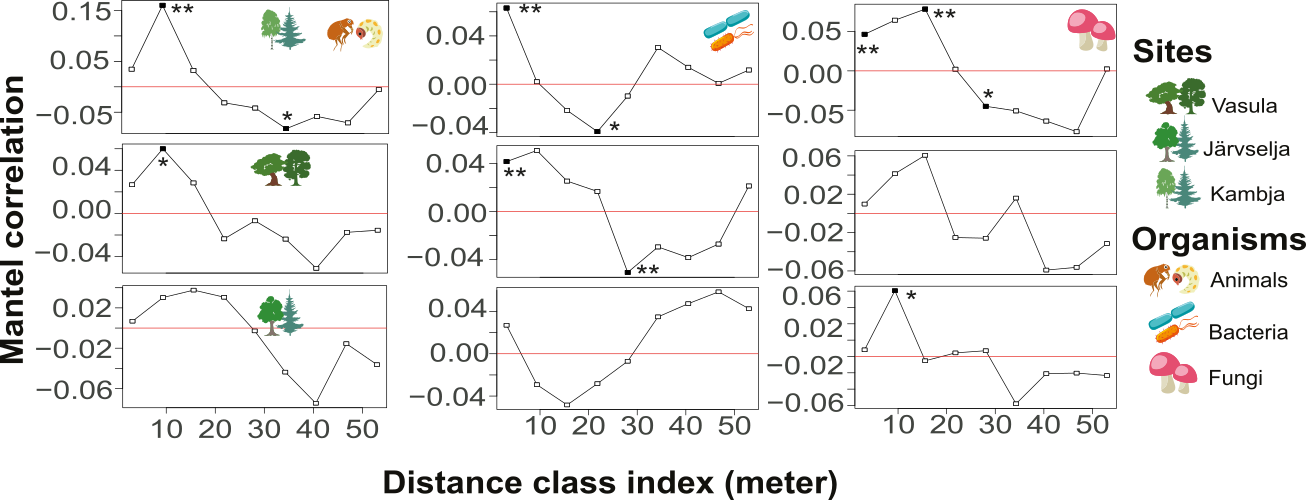


**Figure S3. Spatial Correlation in Communities.** This figure illustrates the spatial autocorrelation for animal, bacterial and fungal communities. Significant correlation values indicate that communities separated by corresponding distance (see X axis) show similarity (positive correlation values) or dissimilarity (negative correlation values). Asterisks highlight statistically significant correlations.
